# Clinical experience with live-attenuated, double-deleted (LADD) listeria monocytogenes targeting mesothelin-expressing tumors

**DOI:** 10.1186/2051-1426-1-S1-P203

**Published:** 2013-11-07

**Authors:** Dirk G Brockstedt, Dung T Le, Raffit Hassan, Aimee Murphy, John Grous, Thomas W Dubensky, Elizabeth M Jaffee

**Affiliations:** 1 Aduro BioTech, Inc., Berkeley, CA, USA; 2 Sidney Kimmel Cancer Center, Johns Hopkins University, Baltimore, MD, USA; 3 National Cancer Institute, Bethesda, MD, USA

## 

Immune-based cancer therapies have demonstrated benefit in multiple clinical trials. The interest in using recombinant bacteria as vaccine vectors for active cancer immunotherapy derives in part from their ability to stimulate multiple innate immune pathways and, at the same time, to effectively deliver antigen for presentation to the adaptive immune system. We have developed a vaccine platform based on LADD Listeria monocytogenes (Lm) in which two virulence genes (actA and inlB) have been deleted from the Lm chromosome which leads to a 1,000-fold attenuation without loss of its ability to induce potent innate and adaptive cellular immunity. Our lead therapeutic, CRS-207, engineered to express the tumor-associated antigen mesothelin, has been tested in 3 clinical studies to date. In a Phase 1 study in 17 patients with advanced cancers that express mesothelin, CRS-207 appeared to be well-tolerated at several dose levels and induced mesothelin-specific CD8+ T cell immunity (Le D Clin. Cancer Res 2012). CRS-207 is currently being tested at a dose level of 1×10^9^ CFU in clinical trials with patients with metastatic pancreatic ductal adenocarcinoma (PDA) and in frontline unresectable malignant pleural mesothelioma (MPM). Importantly, no serious adverse events (AEs) related to CRS-207 have been reported in these studies. In the study with metastatic PDA patients, the most common AEs related to administration of the combination immunotherapy treatment (immunomodulatory doses of cyclophosphamide, GVAX pancreas vaccine and CRS-207) were nausea, vomiting, chills, fatigue, fever and injection site reactions (related to intradermal GVAX injections). No accumulative toxicities in subsequent administrations have been observed. Encouragingly, in this randomized, Phase 2 study, PDA patients receiving the immunotherapy combination of cyclophosphamide, GVAX pancreas vaccine and CRS-207 survived significantly longer than subjects receiving cyclophosphamide and GVAX pancreas vaccine alone. The study was stopped for efficacy at the interim analysis based on recommendation by the independent data monitoring committee. Additionally, in the Phase 1b study in MPM patients where CRS-207 is sequenced with chemotherapy, encouraging anti-tumor activity has been observed, with two minor and two partial responses (PRs) in the first 4 patients. Based on these studies, it appears that CRS-207 can be safely administered intravenously to patients with late-stage metastatic disease at doses up to 1×10^9^ CFU, vaccination can induce the intended immune response and may contribute to extended survival in advanced cancer patients.

